# An implementation of N-way repeated measures ANOVA: Effect coding, automated unpacking of interactions, and randomization testing

**DOI:** 10.1016/j.mex.2020.100947

**Published:** 2020-06-02

**Authors:** Thomas Edward Gladwin

**Affiliations:** a Department of Psychology and Counseling, University of Chichester, Chichester, United Kingdom; b Behavioural Science Institute, Radboud University Nijmegen, Nijmegen, the Netherlands; c Institute for Lifecourse Development, University of Greenwich, London, United Kingdom

**Keywords:** Repeated measures ANOVA, Matlab, M-file, automated, Interactions, Open source, Algorithm, Calculations

## Abstract

The paper presents the details of an implementation of repeated measures ANOVA, consisting of a set of functions to organize data and represent contrasts to be tested and run statistical tests. The implementation is focused on uses common in experimental psychology. An arbitrary number of within-subject factors, each with an arbitrary number of levels, can be used. A non-parametric, randomization- and permutation-based formulation of repeated measures ANOVA was defined and implemented. Methods for testing interactions with categorical and continuous between-subject variables are implemented. Post-hoc tests for exploring interactions are automated. Simulations indicate correct control of false positive rate for all types of test. The software provides output with statistics including *p*-values and partial eta squared.

- An open source implementation of repeated measures ANOVA based on effect coding.
- Generates *p*-values and automatized unpacking of interactions for N-factor designs.
- A non-parametric test is defined based on permutation tests.

## Graphical abstract

**Figure:**
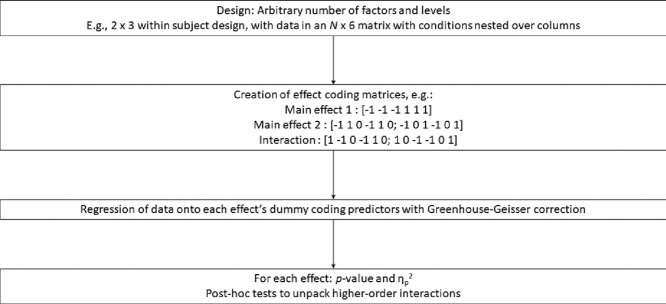

Specifications Table

**Table:** 

Subject Area	Psychology
More specific subject area	*Cognitive psychology, cognitive neuroscience*
Method name	*teg_RMA*
Name and reference of original method	*Not applicable*
Resource availability	*https://github.com/thomasgladwin/teg_RMA*

## Method details

The primary overall aim of the method is to take a matrix of observations, with within-subject factors nested within each other over columns and independent observations on different rows, and a specification of levels of factors, and provide a test of all main effects and interactions. The test can be parametric, via linear regression and Greenhouse-Geisser correction, or non-parametric, via permutation tests. The implementation is in Matlab [15] but should be readily convertible to other languages. The current version is available online as teg_RMA on Github [3], https://github.com/thomasgladwin/teg_RMA. The method has been used, e.g., in psychological studies on anticipatory attentional bias and trial-to-trial history effects on attentional bias variability [5,6].

## Method to create effect coding per effect

The first step of the method is to translate an arbitrary number of factors, each with an arbitrary number of levels, into sets of {−1, 0, 1} effect coding matrices to be used for testing. This is done in the function *teg_RMA_create_ANOVA_dummy*. A vector *levels* is specified that contains, for every factor, the number of levels in that factor. All combinations of levels are generated in a combinations-matrix, with one combination per row. For instance, if the first factor has two levels and the second factor has three levels, then the combinations-matrix will be [1 1 1 2 2 2; 1 2 3 1 2 3]. The effect coding matrices for the main effects are subsequently created based on the combinations-matrix. For a 2 × 3 design, these will be [−1 −1 −1 1 1 1]’ for the 2-level factor and [−1 1 0 −1 1 0; −1 0 1 −1 0 1]’ for the 3-level factor. Note that this representation assumes a nesting of the second factor within the first factor, which must be followed by the columns of the data matrix. The process for generating the effect coding matrices is: for each level *n* after the first, a contrast vector is added to the matrix in which the first level is coded as −1, level *n* is coded as 1, and all other levels are coded as 0. These effect coding matrices provide a basis for all linear contrasts involving the levels of a factor.

Subsequently, similar effect coding matrices are created for all “*tuple*”-way interactions, from 2-way to N-way, where N is the number of factors. For each tuple, a combinations-matrix is again created, now representing all possible combinations of *tuple* factors. For instance, if there are 5 factors, there are 10 possible 3-way interactions (that is, there are 5 over 3 ways of selecting 3 factors out of a set of 5). For each specific interaction, all combinations of the contrast vectors representing the levels of the involved factors are stored in a combinations-matrix. For instance, for an interaction between a 2-level and a 3-level factor, the combinations-matrix would be

$$\begin{array}{cc} 1 & 1\\ 1 & 2 \end{array}$$

The 1′s in the first column represent the single [−1 −1 −1 1 1 1]’ contrast vector of factor 1. The 1 and 2 in the second column represent the two contrast vectors of factor 2. [−1 1 0 −1 1 0; −1 0 1 −1 0 1]’. An effect coding matrix for the interaction is created that contains, for each row of the combinations-matrix, the elementwise-multiplied products of the indicated contrast vectors. In the 2 × 3 example, the 2-way interaction would thus be represented as the effect coding matrix

[1−10−110;10−1−101]′.

## Within-subject tests per main effect and interaction

Subsequently, the effect coding matrices are used, together with the data matrix, to test each main effect and interaction as follows. The core function for this is *teg_RMA_ANOVA*. For each test, the first step is to create a reduced data matrix which only reflects variability due to the combinations of levels relevant to the effect. For instance, when testing the main effect of the 2-level factor in a 2 × 3 design, the raw data matrix with six columns of independently varying values has its data “reduced” to two sets of three columns within which the values per row are the same, and represent the centered difference scores between mean of the last three columns and the mean of the first three columns. This reduction is done by performing a regression separately for each centered (i.e., mean-subtracted) row of the raw data. The vector of scores per row serve as observations, with the effect coding matrix being used as the set of predictors, and the predicted vector replaces the raw data. Thus, variance over cells unrelated to the current test is removed. For instance, for a 2-level factor, each row will have columns consisting of plus and minus some value. This procedure is illustrated in the following example, for one line from a raw data matrix containing the data of a 3 × 2 within-subject design, for which the main effect of the second factor is to be tested:

raw_data_line=[−243−711]

predictor=[−11−11−11]

reduced_data_matrix=[−.667.667−.667.667−.667.667]′

Subsequently, the reduced data matrix is vectorized, each column placed under each other. The vectors of the effect coding matrix are repeated for every participant, so that the −1 / 0 / 1 elements are matched correctly to the vectorized reduced data matrix. For instance, for a 2-level factor and *N* participants, the first *N* values of the vectorized effect coding matrix will be −1, and the second *N* values will be +1.

Finally, all columns are centered, and the data vector is regressed onto the effect coding vectors (note that there is no offset, as the effects are symmetrical around zero for each predictor). If there is only one degree of freedom (i.e., with a 2-level factor or an interaction between 2-level factors), the regression test is performed as normal. This results in the *p*-value of the tested effect via the *F*-test of the ratio of the mean square model (the sum of squares of the regression prediction divided by the model degrees of freedom) and mean square error (the sum of the squares of the regression error divided by the error degrees of freedom). The model degrees of freedom are the number of predictor columns, and the error degrees of freedom are (*N* – 1) * the number of predictor columns, where *N* is the number of participants. Partial eta squared is calculated as the model sum of squares divided by the sum of the model and error sum of squares [10,13].

If the effect coding matrix consists of multiple columns (e.g., the two contrast vectors necessary to test a 3-level factor), Greenhouse-Geisser correction is performed using previously described calculations [1,7]. Epsilon, the measure of violation of the sphericity assumption, is calculated as follows. The vector of eigenvalues, *L*, of the covariance matrix of the reduced data matrix is calculated using Matlab's eig function. Epilson is the square of the sum of the eigenvalues divided by the product of the number of non-zero eigenvalues and the sum of the squared eigenvalues:

epsilon=sum(L).∧2/(N*sum(L.∧2))

The model degrees of freedom and error degrees of freedom were multiplied by epsilon before performing the *F*-test. We note that there are alternative ways to correct for sphericity such as Huynh-Feldt correction [1,^11^,14], for which this code could be adjusted if desired.

For higher-order, *N*-way interactions, follow-up tests are performed in two ways, both aimed at providing a descriptive understanding of what caused a statistically significant interaction. First, the interaction is systematically explored by “unpacking” the interaction: all lower-order, (N-1)-way interactions are performed for each level of the final factor of the interaction, recursively until a single factor is being tested for a specific combination of levels of the other factors involved in the interaction. Second, pairwise t-tests between all conditions defined by all combinations of levels of the factors involved in the higher-order interaction are performed. Because these sets of tests are “protected” by a significant interaction effect and are considered here to have a descriptive purpose not served by losing statistical power, they are not corrected for multiple testing.

## Interactions with between-subject variables

In the above formulation of the analysis, all tests are performed per effect, independently from all other tests. Accordingly, addition of a between-subject variable as specified below does not affect the results of within-subject tests. Note that this approach to tests involving between-subject effects is dissimilar to, e.g., SPSS, and will give slightly different results. The current effect-wise approach was considered preferable for certain experimental goals: first, to remove fluctuations in results that depend on the choice of included variables (although of course other approaches can be taken to deal with this as well), and second, because this approach is conceptually clearly close to common research questions about whether the size of a specific contrast is associated with individual differences rather than the traditional focus on noise-reduction of ANCOVA.

For between-group effects, the between-group factors are transformed into effect coding matrices in the same way as the within-group factors. Interactions with within-subject effects are tested by multiplying the elements of the vectorized effect coding matrix for within-subject effects with the centered contrast scores representing the contrast vectors of the between-group effect coding matrix. Note that centering is essential to avoid group differences from inducing false interaction effects in the presence of a within-subject effect. All resultant pairs of multiplied vectors are used for the final regression, in the same way as for within-subject effects, adjusting the degrees of freedom error given *k* groups to (*N* – *k*) * the number of within-subject predictor columns.

To test interactions with a continuous between-subject variable, *cv*, in a line-by-line way, all participants’ effect coding values were multiplied with their respective centered *cv* value. The same test as for the within-subject effect was then performed to determine whether the within-subject effect depends on the *cv*. Correlations between the *cv* and both pairwise contrasts and individual conditions are reported to explore the cause of a significant result involving an interaction term.

## Non-parametric test

A non-parametric version of the repeated measures ANOVA was implemented as follows. The method is followed as above, up to the final regression. Then, instead of calculating statistics as above, for a user-specified number of iterations (e.g., 10,000) the direction of within-subject effects is randomly, with 50% chance, reversed per participant, thereby preserving the dependencies between columns. This generates samples from a null hypothesis in which the positive versus negative direction of the effect per condition is exchangeable, providing a test of whether the consistency over participants of effects in the observed data is unlikely under this null hypothesis. For interactions with continuous variables, instead of this random reversal of effects, the individual scores in the cv are randomly permuted, removing any systematic relationship between the continuous variable and the within-subject effects. For each iteration the regression is performed and the F-values are stored. This provides a null-hypothesis distribution of F-values. The p-value is defined as the proportion of F-values in the null distribution equal to or greater than the F-value of the unpermuted, observed data.

For further information on such randomization- and permutation-based testing procedures, please see, e.g., Hooton [8], Kennedy [9], Supplementary Materials 1 on the history of the randomization test in Bouwmeester and Jongerling [2], and Pesarin and Salmaso [12].

## Additional information

The current paper describes an implementation of an effect coding approach to repeated measures ANOVA, with independent effect-wise tests, automated drilling-down into interactions, and a non-parametric permutation test for when data do not have an approximately normal distribution. The paper is hoped to be of use to researchers interested either in automated analyses or in open-source code that exposes the details of calculations for understanding or adjustments.

Within-subject effects and interactions involving them are common in, e.g., experimental psychology. It is often the case that researchers wish to work from the perspective of null hypothesis significance testing (NHST). Despite its limitations, NHST plays a valid role in experimental research [4], in which the researcher is often primarily interested in a test of an idea rather than primarily aiming to provide such estimates of population parameters as might be centrally important in, e.g., epidemiology. One essential feature the current method was considered to need was therefore to produce a *p*-value, although an effect size is also produced (and of course, simple mean scores and differences are often perhaps most informative given a theoretical context). Further, the primary interest in the relationship between within-subject effects and between-subject variables was in terms of separate effects; adding between-subject variables should not influence other tests. This led to a “line-by-line” analysis, in which each test was done via a regression analysis specifically generated for that test. This also allowed for a formulation of the method that can be broken down and understood precisely by reasonably statistically informed researchers or students, in terms of effect coding and regression. It also allows variations and improvements to be made where desired or necessary, by breaking in to the method at a point at which all the information necessary for the test is provided, thus possibly efficiently re-using the logistics of organizing data and creating effect coding matrices. It is also envisioned that the software might have educational purposes for students or researchers interested in the “behind the scenes” machinery and computations of within-subject analyses.

Simulations were performed to check whether the implementation correctly recovered simulated effects in the data and controlled for false positives. Initially, ad-hoc simulations were run on a range of designs, with multiple within- and between-subject factors and continuous variables. Within-subject and continuous scores were drawn from a normal distribution (Matlab's randn function). Random between-group assignment was done using the floor function on multiplications of values drawn from the uniform distribution (using the rand function). Dependency between within-subject conditions was created by creating a matrix of random noise, in which for a random selection of half the columns, the column was replaced by the weighted sum of (1 – dep) times itself and dep times the mean of the other columns, the scaling factor dep being the dependency factor. Both the parametric and permutation tests consistently controlled the false positive rate and recovered simulated effects as expected. More simulations were run to check whether false positive rates were controlled for the parametric tests, for a design with 3 × 4 level within-subject factors, 3 × 3 level between-subject factors, and a single continuous between-subject variable. Dependency factors were varied from 0 to 1 in steps of 0.1. For each dependency factor, 25,000 iterations were performed, and the p-values of all tests were stored. The mean p-values over all iterations were used to indicate whether false positive rate was controlled. Over all dependencies and all effects, the worst-case p-value was 0.054, indicating that the false positive rate was controlled for all types of effect. Visual inspection did not suggest any pattern over the p-values related to the degree of dependency.

## Declaration of Competing Interest

The authors declare that they have no known competing financial interests or personal relationships that could have appeared to influence the work reported in this paper.

## References

[1] Abdi H. (2010). The greenhouse-geisser correction. Encyclop. Res. Design.

[2] Bouwmeester S., Jongerling J. (2020). Power of a randomization test in a single case multiple baseline AB design. PLoS ONE.

[3] Gladwin T.E. (2017). *Thomasgladwin/teg_RM*A: Versio*n 1*. Zenodo.

[4] T. Gladwin, Understanding Significance Testing as the Quantification of Contradiction: Conceptual and Practical Foundations of Statistics in the Context of Experimental Research (2020, April 13), doi:10.31234/osf.io/kmrft.

[5] Gladwin T.E., Figner B. (2019). Trial-to-trial carryover effects on spatial attentional bias. Acta Psychol. (Amst).

[6] Gladwin T.E., Vink M. (2020). Spatial anticipatory attentional bias for threat: reliable individual differences with RT-based online measurement. Conscious Cogn.

[7] Greenhouse S.W., Geisser S. (1959). On Methods In The Analysis Of Profile Data. Psychometrika.

[8] Hooton J.W.L. (1991). Randomization tests: statistics for experimenters. Comput. Methods Programs Biomed..

[9] Kennedy P.E. (1995). Randomization Tests in Econometrics. J. Bus. Econ. Statis..

[10] Levine T.R., Hullett C.R. (2002). Eta squared, partial eta squared, and misreporting of effect size in communication research. Hum. Commun. Res..

[11] Maxwell S.E., Delaney H.D. (1990). Designing Experiments and Analyzing data: A model Comparison Perspective.

[12] Pesarin F., Salmaso L. (2010). The permutation testing approach: a review. Statistica.

[13] Richardson J.T.E. (2011). Eta squared and partial eta squared as measures of effect size in educational research. Educat. Res. Rev..

[14] Stevens J.P. (1996). Applied Multivariate Statistics For the Social Sciences.

[15] The Mathworks (2015). MATLAB.

